# Evaluation of the Impact of Probiotic Supplementation on Gingival Inflammation in Subjects Undergoing Orthodontic Treatment with Fixed Appliances or Clear Aligners: A Randomized, Single-Blind Pilot Study

**DOI:** 10.3390/dj13110526

**Published:** 2025-11-10

**Authors:** Jacopo Lanzetti, Serena Queirolo, Giulia Furini Evans, Umberto Gibello, Andrea Deregibus, Francesco Pera

**Affiliations:** Dental School, Department of Surgical Sciences, University of Turin, Via Nizza 230, 10126 Turin, Italy

**Keywords:** fixed orthodontic, clear aligners, oral hygiene, probiotics

## Abstract

**Objectives:** Orthodontic patients are prone to developing gingivitis and require additional tools for maintaining oral hygiene and ensuring plaque control. The present study aimed to evaluate the effects of Bifidobacterium animalis subs. lactis HN019 on plaque accumulation and gingival bleeding in orthodontic patients. **Materials and Methods:** This study is a randomized, single-blind controlled trial. Orthodontic patients were grouped into two groups: subjects undergoing fixed orthodontic treatment and subjects undergoing treatment with a clear aligner. For each group, patients were divided into a test group (probiotic intake) and a control group (no probiotic intake). Patients took the probiotic for one month following a professional oral hygiene session, suspended its intake for 30 days, and then resumed it for another month. Full Mouth Plaque Score (FMPS) and Full Mouth Bleeding Score (FMBS) were collected at all timesteps. **Results:** In fixed orthodontics patients, the results show that the average FMPS improved at every timestep in both groups, especially for the test group, decreasing from 56.20 ± 27.7 to 36.47 ± 23.90. The data show a worsening FMBS during the period when patients do not take the probiotic, from 3.13 ± 3.50 to 6.53 ± 7.42. For the clear aligner patients, the comparison between groups suggests that the plaque index particularly improves for the test group during the period when patients are taking the probiotic, from 43.45 ± 19.52 to 25.93 ± 15.67 and from 26.60 ± 15.79 to 18.93 ± 17.99, respectively. For the bleeding index, data analysis shows a progressive improvement in FMBS at all timesteps in the test group, from 6.93 ± 7.00 to 2.60 ± 2.75. **Conclusions:** The intake of probiotics is useful in decreasing bacterial plaque accumulation and gingival bleeding in orthodontic patients.

## 1. Introduction

Orthodontic patients, both with fixed multibracket appliances or clear aligners, are prone to developing gingivitis [1]. This is due to the fact that patients wearing these devices have difficulty in removing plaque deposits from tooth surfaces, thus inducing superficial and reversible inflammation with a bacterial etiology.

Regarding fixed multibracket appliances, several studies have shown that during orthodontic treatment, brackets attached to the tooth surface cause difficulties related to brushing, resulting in a favorable condition for plaque accumulation. In addition, the more complex the components within a device, the more difficult it will be to clean the tooth surfaces. Plaque build-up can lead to demineralization of the enamel surface as well as to localized gingivitis in 50–70% of patients undergoing fixed orthodontic treatment [2].

Clear aligners represent an alternative to fixed multibracket appliances. These devices, in addition to being more aesthetically appealing, allow for the maintenance of proper oral hygiene in patients undergoing orthodontic treatment. Several studies with 6-month follow-up periods have shown that orthodontic treatment with clear aligners, compared with fixed orthodontic therapy, shows a reduction in plaque accumulation on tooth surfaces while maintaining periodontal health [3,4].

Therefore, orthodontic patients require supplementary oral hygiene tools in addition to mechanical oral hygiene practices. Moreover, there has been increasing demand for alternative active agents for plaque and gingivitis control.

Probiotics, which are defined as ‘living micro-organisms which, when administered in adequate amounts, confer a health benefit on the host’ [5], have been shown to confer various health benefits to humans, including improvements in oral health.

The administration of probiotics, whether as an adjunct to mechanical plaque control or as a single intervention, has produced heterogeneous results. Some studies have shown a benefit of probiotic consumption in reducing the level of gingival inflammation and the accumulation of plaque [6,7], while others have failed to demonstrate any additional clinical benefit [8,9,10].

There are many probiotic strains used in dentistry and as support for oral care [11]. One of these is Bifidobacterium lactis HN019.

HN019 is a strain of the bacterial species Bifidobacterium lactis that is non-pathogenic and safe for human consumption: a 2018 randomized controlled trial found that 10 billion CFU (colony forming units) of B. lactis HN019 administered for one month is safe and well tolerated by the body [12].

Regarding oral health, multiple studies have shown that B. lactis HN019 can be used as an adjunct to a professional oral hygiene session. An in vitro study showed that species of the genus Bifidobacterium can firmly adhere to the subgingival biofilm and significantly reduce Porphyromonas Gingivalis counts [13].

According to a randomized controlled trial, probiotic therapy, as an adjunct to periodontal debridement, showed benefits in analysis at postoperative day 30 and day 90 in patients with periodontal disease [14].

There is also a randomized controlled trial [15] in the literature that confirms the results obtained in the above-mentioned study. These results demonstrate that probiotic therapy improved plaque control, reduced bleeding at the gingival margin (BOMP), and increased the expression of BD-3, TLR4, and CD-4 in periodontal tissues. Probiotic therapy, even when taken for a short period of time, appears to improve the resistance of the oral microbiome to periodontitis risk factors such as plaque accumulation. Furthermore, the test group showed a lower BOMP, which could indicate a slowing of the gingival inflammation process due to probiotic therapy.

Studies on the effects of probiotic use in orthodontic patients are limited, and the findings may be affected by the strains and the duration of probiotics used.

To our knowledge of the literature, this pilot study is the first to evaluate the effects of this probiotic strain, used as an adjunctive treatment for periodontal disease, in orthodontic patients with the goal of improving their oral hygiene.

The aim of this study is to evaluate the effects of Bifidobacterium animalis subs. Lactis HN019 on plaque accumulation and gingival bleeding in patients treated with fixed multibracket appliances and clear aligners. In addition, this study will determine the occurrence of adverse effects due to the intake of a probiotic in orthodontic patients.

## 2. Materials and Methods

### 2.1. Study Design

**Figure 1:**
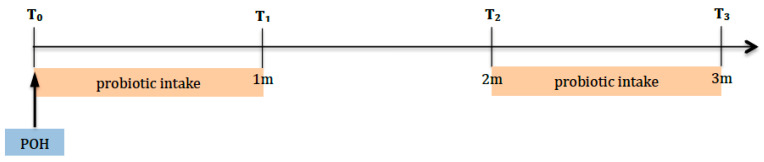
Timeline showing the four time points. T0: Baseline; POH: professional oral hygiene session and probiotic intake was started; T1: after 1 month, probiotic intake was suspended; T2: after 2 months, probiotic intake was continued; T3: after 3 months, the trial ended.

This study was a monocentric, randomized, single-blind (observer) controlled, two-armed trial (Figure 1) and received approval from the Ethics Committee of A.O.U. Città della Salute e della Scienza of Turin (ref. 0001105 of 19 December 2024). The trial was registered on clinicaltrial.gov (NCT06752330). All methods were performed in accordance with the Declaration of Helsinki and the guidelines for good clinical practice. This report was written following the CONSORT guidelines for reporting clinical trials.

### 2.2. Participants

This trial consecutively enrolled patients attending the Orthodontics Unit of C.I.R. Dental School, A.O.U. Città della Salute e della Scienza of Turin (Italy) between January 2025 and June 2025. The sample was selected through the following inclusion and exclusion criteria.

Inclusion criteria:

- Age range of 10 to 30 years old;
- Good general health;
- Patients undergoing fixed multibracket orthodontic treatment;
- Patients undergoing clear aligner orthodontic treatment.

Exclusion criteria:

- Physical, mental or motor disabilities;
- Patients with a diagnosis of periodontal or peri-implant disease (mean pocket depth < 3 mm);
- Patients with a history of periodontal or peri-implant disease;
- Smokers of more than 5 cigarettes per day, including electronic cigarettes;
- Systemic conditions that influence the progression of gingivitis;
- Presence of non-plaque-induced gingivitis;
- Known allergies;
- Use of antibiotics, anti-inflammatories, or probiotics in the 6 months prior to recruitment.

After the recruitment, the patients and their legal guardians were informed about the study. If the patient agreed, they or their legal guardian signed the informed consent form.

Patients were then grouped into two categories: subjects undergoing fixed orthodontic treatment on both arches with edgewise metal orthodontic brackets and subjects undergoing treatment with clear aligners. The patients in each group were further divided into a test group taking the probiotic and a control group not taking the probiotic.

The division into the two groups was performed by following a 1:1 block randomization. An external investigator not involved in the intervention or in the outcome evaluations carried out the randomization via coin toss.

### 2.3. Intervention

The probiotic used in this study was Bifidobacterium lactis HN019, distributed commercially in Italy by Curasept (Curasept Prevent^®^, Curasept S.p.A., Saronno, VA, Italy), with added Kluyveromyces Marxianus Fragilis B0399, colostrum, and biotin. The probiotic was taken by patients following the manufacturer’s directions via 2 chewable tablets daily, morning and evening, after normal daily oral hygiene practices, achieving daily average values of B. lactis HN019 of 2 × 10^7^ CFU and Kluyveromyces marxianus fragilis B0399 of 2 × 10^9^ CFU.

The study was structured into 4 examinations and had a total duration of 3 months (Figure 1): during the first session (T0), after calculating plaque (FMPS) and bleeding (FMBS) indices using the Full Mouth Plaque Score (FMPS), professional oral hygiene treatment was performed.

Each patient was instructed to perform standard home oral hygiene procedures using a medium-bristle manual toothbrush and a fluoride toothpaste (1450 ppm F) without anti-plaque or anti-tartar properties, and to avoid the use of mouthwashes. Patients in the test group were then given a monthly supply of probiotic tablets.

Follow-ups were performed one month (T1), two months (T2), and three months (T3) after the first session.

Probiotic use was suspended between T1 and T2 for 1 month.

The operator who performed the data collection at all time steps was unaware of the patient subgroup (single-blind).

### 2.4. Outcomes

According to a previous study [16], the primary outcome measure was the accumulation of bacterial plaque on the dental surfaces of the entire mouth. The Full Mouth Plaque Score (FMPS) was evaluated in this study. Gingival health was considered a secondary outcome and was assessed through the Full Mouth Bleeding Score (FMBS). FMPS is a dichotomous index indicating the presence of bacterial biofilm on 6 tooth surfaces: the mesio-vestibular, vestibular, disto-vestibular, disto-palatal/lingual, palatal/lingual, and mesio-palatal/lingual surfaces. FMBS is a dichotomous index indicating the presence of gingival bleeding on 6 surfaces, as with FMPS, after light probing. The ratio of surfaces with plaque or bleeding to the total number of assessed surfaces is presented as a percentage [17].

The visual examination was performed under standardized conditions with standardized illumination and using a dental mirror and an XP23/UNC 15 periodontal probe (Hu-Friedy, Chicago, IL, USA). The examination was performed by the same experienced and calibrated dental hygienist (Kappa score 0.906; 95% confidence interval [CI] 0.895 to 0.997; *p* < 0.01), with the help of an erythrosine-based plaque detector (Mira-2-Ton Solution, Hager & Werken GmbH & Co., Duisburg, Germany) at 2.5× magnification. The operator performing the data collection was unaware of the group to which the evaluated patient belonged.

Compliance was rated “good” when a patient forgot to take 3 or fewer tablets per week. If the patient reported any adverse events during the session, this is properly documented and the patient was advised to stop taking the probiotic. The assessment of adverse effects related to the use of the probiotic was performed during each follow-up session through clinical examinations and interviews with the patient.

### 2.5. Statistical Analysis

The sample size was assessed using Jamovi (version 2.3.28.0) and was calculated based on a previous study [16] with a confidence level of 95%, a type I error of 5%, and an effect size of 0.80, and FMPS change was considered the primary outcome variable, considering a difference of 20% in terms of the frequency of plaque-positive sites between the two treatment groups as clinically relevant. Therefore, 26 patients were required for each experimental group, totaling 52 patients. In total, 60 patients were recruited.

The data was collected using a spreadsheet in Microsoft Excel (version 14.4.9). Statistical analysis was performed using Jamovi (version 2.3.28.0). The Kolmogorov–Smirnov normality test was used to verify the normal distribution of data. For the comparison of the clinical indices between the two groups, Student’s *t*-test for independent samples was used and *p*-values < 0.05 were considered statistically significant.

## 3. Results

### 3.1. Population Characteristics

A total of 60 patients were enrolled in this study. Of these, 30 were treated with a fixed appliance and 30 were treated with a clear aligner. The demographic characteristics of the entire sample, divided between patients with fixed appliances and those with clear aligners, are shown in Table 1. No statistical differences were observed between the two groups.

None of the patients in the test group reported adverse effects due to the use of probiotics, and no clinical alterations attributable to their administration were observed.

For each group, patients were randomized into two groups of 15 patients each.

At baseline (T0), there were no differences in clinical indices in the two groups of patients with fixed orthodontic appliances (Table 2).

Similarly, there were no differences in clinical outcomes in patients with clear aligners at baseline (Table 3).

### 3.2. Fixed Orthodontics Patients

**Figure 2:**
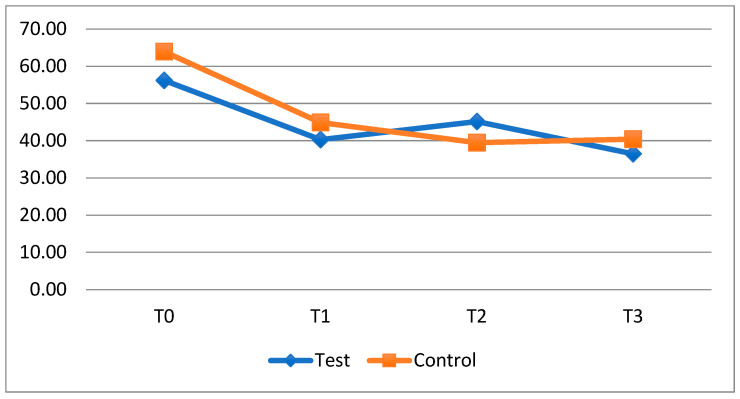
Trend of the average FMPS of the fixed orthodontics patients divided into the test and control groups.

The data collected showed that the average FMPS improved at every timestep in both groups (Figure 2), especially for the test group, where there was an improvement between T0 and T1 (from 56.20 ± 27.70 to 40.27 ± 26.90) and between T2 and T3 (from 45.13 ± 24.60 to 36.47 ± 23.90), i.e., the periods when the probiotic was taken, but not between T1 and T2, the period of non-intake, in which there was a slight worsening of the plaque index (from 40.27 ± 26.90 to 45.13 ± 24.60).

The comparison between groups at the different timesteps does not reveal any statistically significant differences with regard to FMPS (Table 4), but a worsening of plaque accumulation can be observed in the test group during the period of not taking the probiotic (T1–T2: 4.87 ± 20.8).

**Figure 3:**
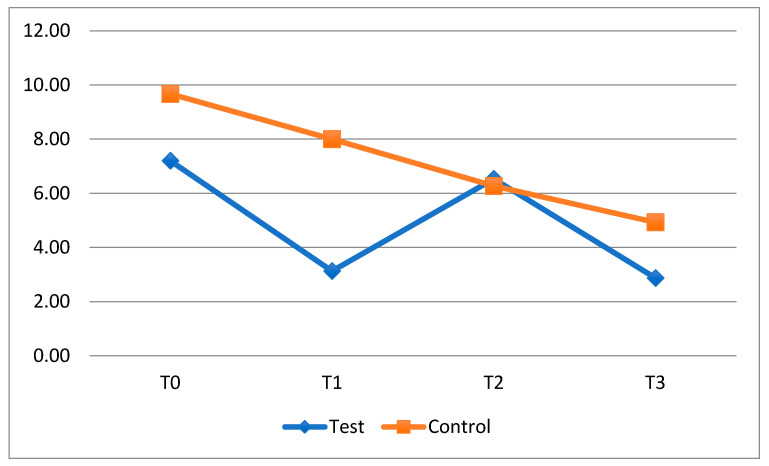
Trend of the average FMBS of the fixed orthodontics patients divided into the test and control group.

As regards the results obtained with for FMBS index, it was possible to carry out an analysis quite similar to that carried out for the FMPS (Figure 3). As with the FMPS, it was possible to observe an average improvement in the bleeding index between T0 and T1 (from 7.20 ± 9.70 to 3.13 ± 3.50) and between T2 and T3 (from 6.53 ± 7.42 to 2.87 ± 4.27) in the group exposed to the probiotic. Meanwhile, the data showed a worsening of FMBS in the period when patients did not take the probiotic. In particular, the test group showed an important worsening of the bleeding score in the T1-T2 period (3.40 ± 6.68; *p* = 0.051).

The comparison between groups at different timesteps does not reveal any statistically significant differences with regard to FMBS (Table 5).

### 3.3. Clear Aligner Patients

For the clear aligner patients, the comparison between groups at different timesteps does not reveal any statistically significant differences with regard to FMPS (Table 6), but the results do suggest that the plaque index particularly improves for the test group during the periods when patient were taking the probiotic: T0-T1 (from 43.45 ± 19.52 to 25.93 ± 15.67) and T2-T3 (from 26.60 ± 15.79 to 18.93 ± 17.99). In fact, the test group displayed a greater reduction in FMPS than the control group between T0 and T1 (−17.53 ± 12.32; *p* = 0.003) and between T2 and T3 (−7.67 ± 9.32; *p* = 0.009).

**Figure 4:**
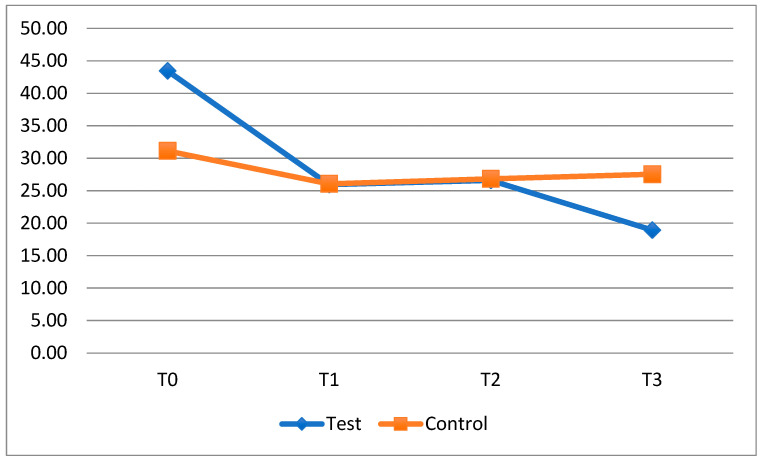
Trend of the average FMPS of the clear aligner’s patients divided into the test and control group.

The trend of the average FMPS for the clear aligner patients is shown in Figure 4.

**Figure 5:**
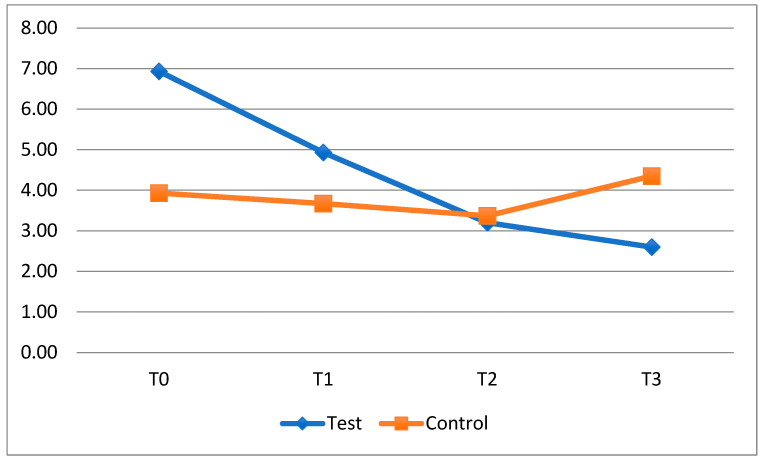
Trend of the average FMBS of the clear aligner patients divided into the test and control group.

Regarding the bleeding index, the statistical analysis performed showed no statistically significant differences. However, data analysis showed a progressive improvement in FMBS at all timesteps in the test group (Figure 5), especially at T3, while patients in the control group experienced a smaller improvement and, at T3, displayed an increase in gum bleeding (Test: 2.60 ± 2.75; control: 4.35 ± 4.97; *p* = 0.243) (Table 7).

## 4. Discussion

Orthodontic patients, especially those with fixed multibracket appliances, need to be constantly monitored during treatment, as the presence of orthodontic structures might hinder the proper cleaning of tooth surfaces.

The results of this study indicate that the presented probiotic therapy could benefit professional oral hygiene sessions in orthodontic patients with fixed appliances or clear aligners, as the test subgroups—who was administered the probiotic—showed decreasing levels of plaque accumulation and gingival bleeding when patient were taking the probiotic. However, these decreases were not statistically significant.

From the data obtained, a reduction in plaque accumulation between T0 and T1 in the exposed subgroups emerged both in patients with fixed orthodontic appliances and in patients with clear aligners.

When the probiotic was suspended, between T1 and T2, plaque levels in exposed subjects tended to rise, reaching the level of the control subgroups, especially in patients with fixed orthodontics. In the next time interval (T2–T3), with the resumption of probiotics, there was again a decrease, which was not statistically significant, in average levels of FMPS.

Meanwhile, the groups of patients who did not take HN019, following a reduction in the mean plaque index in the first observation period (T0-T1), showed a stable trend regarding this outcome in subsequent timesteps (T1–T2 and T2–T3).

With regard to the bleeding index, the collected data show, in the period T0–T1, a greater decrease in the bleeding index in the test subgroups with fixed orthodontics appliances, but this result is not statistically significant. As for plaque, at the suspension of the probiotic (T1–T2), the FMBS rises, slightly above the score for the control subgroups in the same period, and then decreases in a not statistically significant way in the last observation interval, when taking the probiotic again. This finding is interesting because it suggests that in order to maintain stable gingival inflammation in a patient with a fixed appliance, it might be useful to supplement standard home oral hygiene practices with the intake of HN019.

In the control groups, the average FMBS followed a similar course to the plaque index: it was reduced at T1 and then remained stable in subsequent timesteps.

Other trials [18,19], which studied the efficacy of two probiotics on *S. mutans* reduction in plaque taken from the tooth surface of fixed orthodontics patients, also found no statistically significant differences.

Another study [20] looked at the count of periodontopathogenic bacteria and showed how two weeks of Lactobacillus reuteri intake reduces the amount of *Porphyromonas* sp., *Treponema* sp. and *Prevotella* sp. and is correlated with a 50% reduction in the OHI-S index (Plaque index).

A recent literature review [21] stated that along with common recommendations regarding home oral hygiene practices, probiotics can also be recommended to control cariogenic bacteria, reducing the risk of white spots and caries, and also to effectively manage gingival biofilms and periodontal pathogens, emphasizing the effectiveness of probiotics in effectively reducing the count of pathogenic bacteria, such as *P. gingivalis*, in dental plaque and saliva samples.

However, the same review concluded by asserting that the selected studies show conflicting clinical and microbiological results.

Such discrepancies may be due to the different methods, dosage regimens, durations of probiotic prescriptions, and clinical and biological measurements being used.

An umbrella review [22] suggested that the administration of probiotics may be effective in improving or maintaining oral health in patients treated with fixed orthodontic devices.

This study demonstrated that, as highlighted in the literature, there is no clear evidence of the effect of using probiotics on oral health, and cited two reviews with conflicting conclusions [23,24], underlining the moderate bias risk of the studies included in this review.

The probiotic used in this study had a greater adjuvant effect in decreasing the FMPS and FMBS indexes for patients treated with fixed appliances, providing oral benefits. In the case of clear aligners, this phenomenon is less pronounced as the baseline situation is better than that of patients with fixed multibracket appliances and it is easier to thoroughly clean tooth surfaces during the home oral hygiene routine. Therefore, if we consider patients with clear aligners as low-risk patients, the use of probiotics does not provide any benefit, as shown by a study evaluating the administration of 3 mg of Lactobacteria and 2 mg of Glycobacteria on Mutans Streptococci and Lactobacilli counts [25]. Contrary to the study by Chen et al. [26], our study shows an improvement in the plaque index during the periods when the clear aligner patients took the probiotic (T0–T1 and T2–T3). The use of HN019 had a significant impact on dental plaque accumulation in exposed patients, indicating a possible benefit in oral hygiene during orthodontic treatment with clear aligners.

The same result can be observed by analyzing the FMBS after the second period of application (T3), when the test subgroup with clear aligners displayed a decrease in the bleeding index, while in the control subgroup, the same index increased. The difference between these subgroups, however, was not statistically significant. The reduction in the bleeding index at the last timestep suggests that the probiotic could play a crucial role in counteracting gingival inflammation during orthodontic treatment with clear aligners, improving the patient’s oral condition.

The greater effectiveness of probiotic administration in patients wearing clear aligners may be due to the fact that the aligner allows a “closed microenvironment” to form on the tooth surfaces, making it easier for probiotics to temporarily colonize and compete with pathogenic bacteria.

Furthermore, clear aligners eliminate the need for brackets, wires, or retention niches, which can promote plaque formation. In these cases, probiotics are more likely to interfere with the developing biofilms, making their anti-plaque effect more evident.

No patients enrolled reported any adverse or undesirable reactions to the probiotic, such as bloating, flatulence and abdominal cramps or, in more serious cases, diarrhea or constipation. This demonstrated the safety of therapy with B. lactis HN019. In general, probiotic intake is safe for patients; indeed, a randomized phase 2 trial [27] published in 2020 proved that 21 days of treatment with L. brevis reduced the occurrence of traumatic oral lesions due to fixed appliances in 2.5 days on average. The total number of days with oral lesions was 4.9 in the placebo group and 2.5 in the test group. In addition, patients who used the probiotic reported lower pain levels.

The safety of probiotic therapy is also evidenced by a double-blind RCT [27], indicating good compliance and adherence to treatment by adolescents wearing orthodontic appliances.

Thus, the probiotic Bifidobacterium lactis HN019 can be used as an aid in restoring the balance of the oral ecosystem in this type of patient, especially considering the ease of following the treatment.

### 4.1. Limitations of the Study

In this study, the duration of 3 months provided adequate time for probiotics to settle in the oral cavity and provide a benign effect. On the other hand, a limitation of the study was that it did not consider a specific plaque index for the patients wearing fixed orthodontics, such as the Orthodontic Plaque Index (OPI) [28].

Another limitation of this study is the sample size, which is too small. Despite the calculations performed, the statistical power may not be sufficient to detect clinically significant differences.

Furthermore, age, compliance, type of malocclusion, and type of orthodontic treatment are factors that could influence the patient’s oral hygiene habits. All of these variables constitute potential limitations to the study performed. Although the differences in the variables between the two groups were not statistically significant at baseline, a more robust randomization method, such as computerized or stratified randomization, could have been used.

In this study, Student’s *t*-test for independent samples was used at multiple time points. However, longitudinal data analysis could have avoided Type I error inflation, making a temporal analysis more appropriate.

### 4.2. Recommendations for Future Research

The results obtained from this research stimulate further investigation into the use of probiotics for the maintenance of oral hygiene in orthodontic patients. Future research is necessary to identify the action of probiotics in the oral ecosystem, potentially in combination with chlorhexidine- or fluorine-based devices to increase the efficacy of probiotic therapy.

In future research, it would be interesting to determine the long-term effects of probiotics in orthodontic patients and evaluate their effectiveness in the prevention of white spots or new dental caries and to highlight the potential beneficial role of probiotics as adjuvants for home care in patients undergoing fixed orthodontic therapy.

## 5. Conclusions

The results of this study indicate that the intake of Bifidobacterium animalis subsp. lactis HN019 significantly reduces bacterial plaque accumulation and gingival bleeding in orthodontic patients, particularly those undergoing clear aligner treatment. These findings support the potential role of probiotic supplementation as an effective adjunct to conventional oral hygiene measures in orthodontic care.

## Figures and Tables

**Table 1:** Demographic characteristics of the entire sample.

	Fixed (N = 30)	Clear Aligner (N = 30)	Total (N = 60)	*p* Value *
Gender [n (%)]				0.605
Female	15.0 (50.0%)	17.0 (56.7%)	32.0 (53.3%)	
Male	15.0 (50.0%)	13.0 (43.3%)	28.0 (46.7%)	
Age [mean (SD)]	18.9 (5.3)	19.2 (5.0)	19.1 (5.1)	0.784
Malocclusion [n (%)]				0.579
Agenesis	5.0 (16.7%)	6.0 (20.0%)	11.0 (18.3%)	
Class II	9.0 (30.0%)	13.0 (43.3%)	22.0 (36.7%)	
Class III	7.0 (23.3%)	4.0 (13.3%)	11.0 (18.3%)	
Cross bite	1.0 (3.3%)	0.0 (0.0%)	1.0 (1.7%)	
Crowding	5.0 (16.7%)	6.0 (20.0%)	11.0 (18.3%)	
Deep bite	2.0 (6.7%)	0.0 (0.0%)	2.0 (3.3%)	
Open bite	1.0 (3.3%)	1.0 (3.3%)	2.0 (3.3%)	
Extraction [n (%)]				1.000
No	23.0 (76.7%)	23.0 (76.7%)	46.0 (76.7%)	
Yes	7.0 (23.3%)	7.0 (23.3%)	14.0 (23.3%)	
Pocket Depth [mean (SD)]	2.3 (0.6)	2.5 (0.6)	2.4 (0.6)	0.207

* Comparison between the fixed and clear aligner groups with Pearson’s chi-squared test and linear-model ANOVA.

**Table 2:** Demographic characteristics and clinical parameters of the patients with fixed orthodontic appliances.

	Test (N = 15)	Control (N = 15)	Total (N = 30)	*p* Value *
Gender [n (%)]				0.068
Female	5.0 (33.3%)	10.0 (66.7%)	15.0 (50.0%)	
Male	10.0 (66.7%)	5.0 (33.3%)	15.0 (50.0%)	
Age [mean (SD)]	19.1 (4.3)	18.6 (6.3)	18.9 (5.3)	0.789
FMPS T0 [mean (SD)]	56.2 (27.7)	63.9 (25.1)	60.1 (26.3)	0.430
FMBS T0 [mean (SD)]	7.2 (9.7)	9.7 (9.6)	8.4 (9.6)	0.491

* Comparison between the test (with probiotics) and control (no probiotics) groups with Pearson’s chi-squared test and linear-model ANOVA.

**Table 3:** Demographic characteristics and clinical parameters of the patients with clear aligners.

	Test (N = 15)	Control (N = 15)	Total (N = 30)	*p* Value *
Gender [n (%)]				0.713
Female	8.0 (53.3%)	9.0 (60.0%)	17.0 (56.7%)	
Male	7.0 (46.7%)	6.0 (40.0%)	13.0 (43.3%)	
Age [mean (SD)]	19.3 (5.9)	19.1 (4.1)	19.2 (5.0)	0.915
FMPS T0 [mean (SD)]	43.5 (19.5)	31.1 (17.0)	37.3 (19.0)	0.076
FMBS T0 [mean (SD)]	6.9 (7.0)	3.9 (3.4)	5.4 (5.6)	0.147

* Comparison between the test (with probiotics) and control (no probiotics) groups with Pearson’s Chi-squared test and Linear Model ANOVA.

**Table 4:** Fixed orthodontics patients: comparison of FMPS between groups at different timesteps.

	Group	N	Mean (SD)	*p* Value *
FMPS T0	Test	15	56.20 (27.7)	0.430
	Control	15	63.93 (25.09)	
FMPS T1	Test	15	40.27 (26.9)	0.623
	Control	15	44.87 (23.60)	
FMPS T2	Test	15	45.13 (24.6)	0.518
	Control	15	39.47 (22.81)	
FMBS T3	Test	15	36.47 (23.9)	
	Control	15	40.40 (24.70)	
FMBS T0-T1	Test	15	−15.93 (31.7)	0.751
	Control	15	−19.07 (20.68)	
FMBS T1-T2	Test	15	4.87 (20.8)	0.086
	Control	15	−5.40 (8.12)	
FMBS T2-T3	Test	15	−8.67 (20.2)	0.192
	Control	15	0.93 (19.13)	

* Comparison between the test (with probiotics) and control (no probiotics) groups with Student’s *t*-test for independent samples.

**Table 5:** Fixed orthodontics patients: comparison of FMBS between groups at different timesteps.

	Group	N	Mean (SD)	*p* Value *
FMBS T0	Test	15	7.20 (9.70)	0.491
	Control	15	9.67 (9.65)	
FMBS T1	Test	15	3.13 (3.50)	0.092
	Control	15	8.00 (10.22)	
FMBS T2	Test	15	6.53 (7.42)	0.930
	Control	15	6.27 (8.94)	
FMBS T3	Test	15	2.87 (4.27)	0.279
	Control	15	4.93 (5.85)	
FMBS T0-T1	Test	15	−4.07 (7.49)	0.529
	Control	15	−1.67 (12.50)	
FMBS T1-T2	Test	15	3.40 (6.68)	0.051
	Control	15	−1.73 (7.13)	
FMBS T2-T3	Test	15	−3.67 (8.37)	0.346
	Control	15	−1.33 (4.32)	

* Comparison between the test (with probiotics) and control (no probiotics) groups with Student’s *t*-test for independent samples.

**Table 6:** Clear aligner patients: comparison of FMPS between groups at different timesteps.

	Group	N	Mean (SD)	*p* Value*
FMPS T0	Test	15	43.45 (19.52)	0.076
	Control	15	31.13 (17.00)	
FMPS T1	Test	15	25.93 (15.67)	0.982
	Control	15	26.07 (15.93)	
FMPS T2	Test	15	26.60 (15.79)	0.972
	Control	15	26.82 (19.05)	
FMPS T3	Test	15	18.93 (17.99)	0.214
	Control	15	27.54 (19.06)	
FMPS T0-T1	Test	15	−17.53 (12.32)	**0.003**
	Control	15	−5.07 (8.78)	
FMPS T1-T2	Test	15	0.67 (13.31)	0.983
	Control	15	0.75 (10.04)	
FMPS T2-T3	Test	15	−7.67 (9.32)	**0.009**
	Control	15	0.713 (6.87)	

* Comparison between the test (with probiotics) and control (no probiotics) groups with Student’s *t*-test for independent samples.

**Table 7:** Clear aligner patients: comparison of FMBS between groups at different timesteps.

	Group	N	Mean (SD)	*p* Value *
FMBS T0	Test	15	6.93 (7.00)	0.147
	Control	15	3.93 (3.39)	
FMBS T1	Test	15	4.93 (6.58)	0.520
	Control	15	3.67 (3.66)	
FMBS T2	Test	15	3.20 (3.05)	0.882
	Control	15	3.37 (3.27)	
FMBS T3	Test	15	2.60 (2.75)	0.243
	Control	15	4.35 (4.97)	
FMBS T0-T1	Test	15	−2.00 (6.37)	0.372
	Control	15	−0.27 (3.75)	
FMBS T1-T2	Test	15	−1.73 (5.04)	0.319
	Control	15	−0.29 (2.19)	
FMBS T2-T3	Test	15	−0.60 (1.68)	0.098
	Control	15	0.98 (3.14)	

* Comparison between the test (with probiotics) and control (no probiotics) groups with Student’s *t*-test for independent samples.

## Data Availability

The original contributions presented in this study are included in the article. Further inquiries can be directed to the corresponding author.
